# Inner ear organoids: Recent progress and potential applications

**DOI:** 10.1016/j.fmre.2023.07.013

**Published:** 2023-12-05

**Authors:** Yiyun Lou, Yaoqian Liu, Mingxuan Wu, Gaogan Jia, Mingyu Xia, Wenyan Li

**Affiliations:** a ENT Institute and Otorhinolaryngology Department of Eye & ENT Hospital, State Key Laboratory of Medical Neurobiology and MOE Frontiers Center for Brain Science, Fudan University, Shanghai 200031, China; b NHC Key Laboratory of Hearing Medicine, Fudan University, Shanghai 20003, China

**Keywords:** Inner ear, Organoid, Stem cell, Hair cell, 3D culture

## Abstract

The inner ear is one of the most complicated structures that harbor organs for the perception of sound and balance, which is deep in the temporal bone and challenging to operate. Organoids serve as promising platforms for understanding developmental processes and pathological dysfunctions and discovering therapeutic drugs and gene therapy strategies for disorders in the inner ear. To better understand the origin and application value of organoids, we reviewed the developmental history and advancement of inner ear organoid research. We summarized the cell sources of organoids and the matrices supporting their formation. The history of the research on inner ear organoids derived from pluripotent stem cells (PSCs) and primary progenitor cells has been clarified in detail. We elaborated on the applications of organoids in inner ear development, hereditary deafness modeling, and hair cell (HC) regeneration strategy formulation. Finally, we mentioned limitations of the current culture methods and applications of inner ear organoids, and described several prospects for optimizing next-generation organoids of the inner ear for potential translational applications.

## 1 Introduction

Harbored in the inner ear, the cochlea and vestibule are the two critical functional organs responsible for hearing and balance perception. Mechanosensory hair cells are the sensors that transform external stimuli into electrical biosignals, which are then relayed by spiral ganglion and vestibular ganglion neurons. The auditory cortex/vestibular nucleus receives afferent electrical signals along the neural pathway and finally perceives hearing or balance [1,2]. Hair cells (HCs) and sensory neurons in the inner ear are vulnerable to risk factors such as genetic defects, noise exposure, ototoxic drugs, and the aging process [3,4]. In mammals, the impairment of these two key cell types leads to permanent hearing loss and vertigo due to the limited regenerative capacity of the HCs and neurons [5]. It is estimated that more than 1.5 billion people worldwide have diverse hearing loss, and ∼30% need clinical treatment [6]. Likewise, 7.4% of the population reported a lifetime disorder of vestibular vertigo [7]. Thus, it is urgent to develop practical strategies to recover and reestablish inner ear sensory functions for these people.

Advanced bio-techniques such as regeneration medicine [8] and gene therapy [9] bring new hopes for sensory reconstruction and recovery of the inner ear. Currently, the progress of functional regenerative strategies is still limited, primarily due to the lack of pathological and physiological models that are reliable and effective for testing these strategies before successful applications in the clinic. Traditional *in vitro* animal tissue/cellular models of the inner ear exhibit various limitations, such as morphological differences [10], low efficiency [11], and large genome gaps [12]. For *in vivo* experiments, access to inner ear tissues without irreversible damage to the surrounding structure is technically challenging, and noninvasive imaging approaches cannot provide sufficiently intuitive information [13,14]. Furthermore, ethical approval for the use of continuous human samples is difficult. To address these issues, high demands for inner ear research using modified substitutive models with sustainable, accessible, and highly simulative properties are highlighted. As a result, organoids serve as powerful models for inner ear research and have drawn significant attention.

Unlike cells in the traditional two-dimensional (2D) culture system, inner ear organoids are developed from stem/progenitor cells based on three-dimensional (3D) culture technology and resemble the genetic profiles, cell types, structure, and function of inner ear tissues [15,16]. The 3D culture system provides an extracellular environment imitating physiological conditions, which induces stem cells/progenitor cells to proliferate and differentiate into organ-specific cell types under specific factors [17,18]. For the first time, inner ear organoids serve as an unparalleled tool to observe the dynamic process of inner ear development and rebuild functionalized human HCs and neural connections *in vitro* [12,19]. Recently, researchers have attempted to apply inner ear organoids derived from stem cells/progenitors to address neuropathological defects and screen drugs that can prevent ototoxicity or promote HC regeneration [20,21]. The construction and application of inner ear organoid models have led to new ideas and models for diagnosing and treating vestibular vertigo and sensorineural hearing loss.

In this review, we briefly summarize the progress of organoids, including cell sources and culture strategies, and describe the historical overview and latest applications of inner ear organoids. Furthermore, the deficiency and technical limitations of inner ear organoid models are also described. We identify several prospective ways for optimizing inner ear organoids and potential clinical applications of inner ear organoids.

## 2 Origin and matrices for organoids

Organoids are defined as complex 3D structures consisting of organ-specific cell types and recapitulating parts of the organ function. In addition, they are derived from stem cells or organ progenitor cells and self-organize based on the spatial support from 3D substrates [22,23]. Ever since pioneering studies on cortical organoids derived from embryonic stem cells [24] and intestinal organoids derived from single adult stem cells (ASCs) [25] were constructed, there has been a robust evolution of organoids in recent decades. Organoids are promising platforms for modeling and investigating tissue development, homeostasis, and regeneration.

### 2.1 The original cell types for organoids

**Fig. 1:**
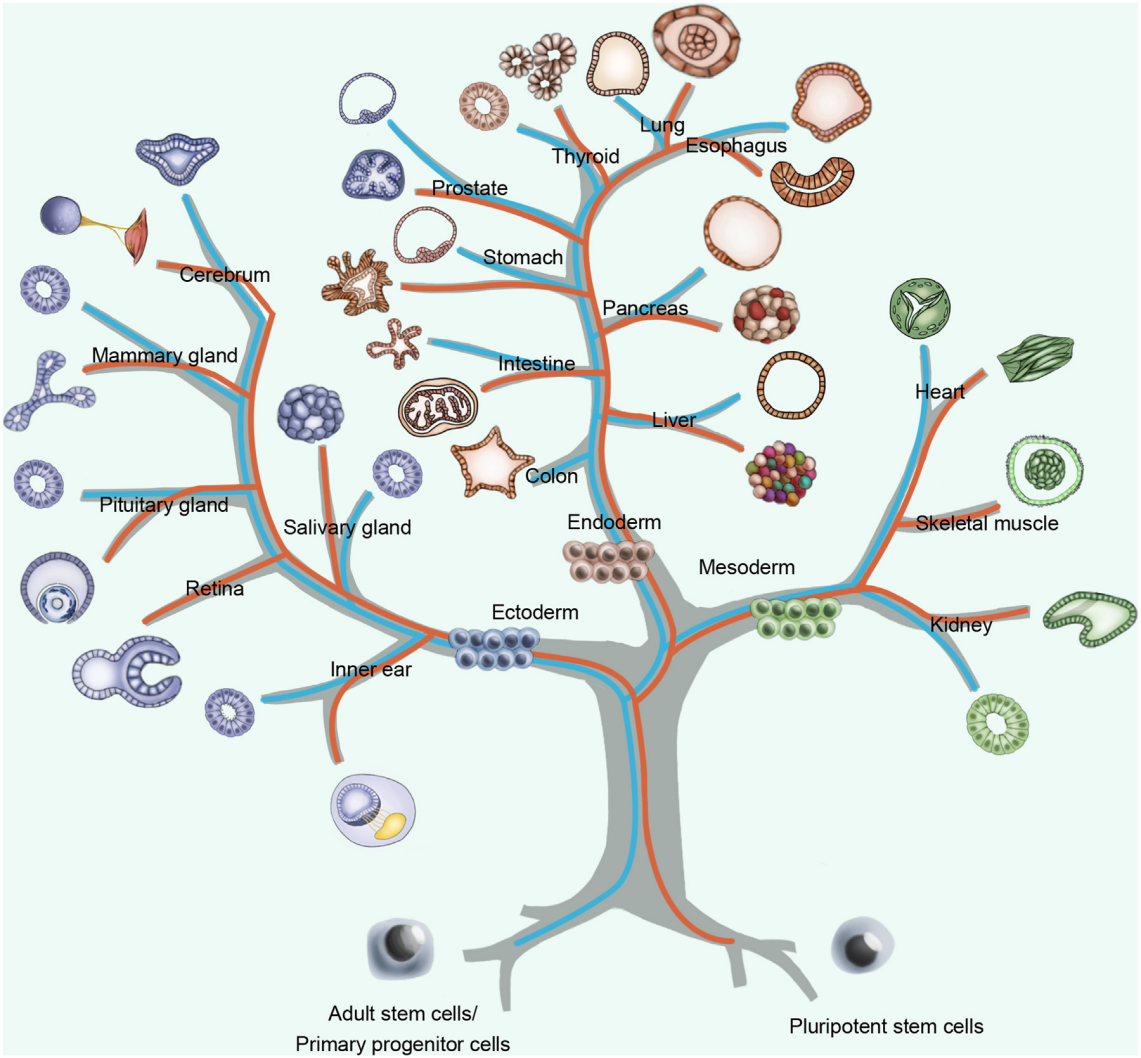
**Various organoids were established, resembling corresponding organs.** Organoids from pluripotent stem cells (PSCs, red line) and adult stem cells/primary progenitor cells (ASCs/PPCs, blue line) resemble target organs or tissues from ectoderm, endoderm, and mesoderm have been developed. Most organoids can be derived from both ASCs/PPCs and PSCs.

Generally, organoids can be derived from two types of cells: pluripotent stem cells, including pluripotent embryonic stem cells (ESCs)/induced pluripotent stem cells (iPSCs); and organ-restricted adult stem cells (ASCs)/primary progenitor cells (PPCs) [22,26] (Fig. 1). PSC-derived organoids form structures through a process that highly recapitulates primary organ development from the original embryonic stage. As a result, they generally possess complex structures and contain various cell types, including mesenchymal, epithelial, and even endothelial components. PSCs expand and differentiate toward each germ layer (endoderm, mesoderm, and ectoderm) and subsequently differentiate into the target tissue through a specific stepwise protocol. Taking liver organoids as an example, activin A was used to activate transforming growth factor beta (TGF-β) signaling to generate definitive endoderm, and fibroblast growth factor-10 (FGF10), basic fibroblast growth factor (bFGF) and bone morphogenetic protein 4 (BMP4) were then applied to induce a hepatic fate [27]. In addition to hepatic organoids, PSC-derived organoids mimic target organs, including those from the ectoderm (inner ear, retina, cerebra, etc.), endoderm (small intestine, stomach, lung, etc.), and mesoderm (heart, skeletal muscle, kidney, etc.) [28].

While ASCs/PPCs have lower variability, which determines their ability to stably differentiate into tissue-specific cells during physiological tissue self-renewal or regeneration *in vivo*. Accordingly, ASC/PPC-derived organoids have a higher degree of directional specificity but less complex structures and functions than those derived from PSCs [26]. For instance, ASC-derived intestinal organoids contain only the intestinal epithelium and lack niche-specific mesenchyme, immune cells, vascularization, innervation, or a microbiome [29]. Under cocktail-based conditions, ASC-derived intestinal organoids could be self-organized without stromal and mesenchymal cells. Conventionally, the Wnt signal amplifier R-spondin-1, BMP inhibitor Noggin, and epidermal growth factor (EGF) stimulate intestinal stem cell proliferation [25,30]. A combination of the Wnt agonist CHIR99021 (CHIR) and the Notch activator valproic acid (VPA) further enhanced the self-renewal of cultured organoids, resulting in high-purity Lgr5+ intestinal stem cells. Lgr5+ stem cells were then directed to multiple mature intestinal cell type lineages, including absorptive enterocytes and secretory goblet/Paneth cells, by modulating Wnt and Notch signaling [31]. By modifying cocktails of growth factors and cell isolation procedures, similar protocols were used to develop general and cancerous organoids from other organs, including the liver, pancreas, colon, salivary gland, fallopian tube, mammary gland, prostate, airway, and kidney [28].

### 2.2 3D matrices for organoid culture

Apart from the soluble culture medium containing essential growth factors and compounds, another prerequisite for organoid culture is the microenvironment provided by the 3D matrices. In general, 3D matrices mimic the biological and mechanical properties of the *in vivo* extracellular matrix (ECM), which provides spatiotemporal support for cellular structure [32]. Thus, the 3D microenvironment has attracted significant attention for organoid culture [33], and various matrices for 3D organoid culture have been developed [34]. Natural hydrogels, compound matrices composed of crosslinked polymers containing water and multiple solutes, are ideal candidates in favor of the 3D culture of organoids due to their accordance with the biological and physiochemical characteristics of ECM [35,36]. Matrigel is a typical tumor-derived natural hydrogel featuring raw and complex compositions, which determines defects of uncontrollable variation between batches and potential risks of immunogenicity or tumorigenicity [37]. Recently, decellularized tissue-derived hydrogels were developed. Compared with Matrigel, they have the advantages of upregulating tissue-specific genes and more amounts and subtypes of collagens [38], [39], [40], [41].

However, the complex, undefined, highly variable constituents of natural hydrogels fail to optimize organoids with precise characteristics, such as tunable stiffness, elasticity, flexibility, and durability. As a result, mechanically tunable synthetic hydrogels were proposed as optimization strategies to be developed. Stiffness is an important mechanical cue that regulates cell behavior and differentiation fate. It has been reported that stiffness modification can be achieved by altering the molecular weight of the monomer, polymer density, and distance between functional groups in the mesh-like structure [34]. For example, by changing the concentration of gelatin-hydroxyphenyl propionic acid (Gtn-HPA), medium-stiffness Gtn-HPA hydrogels were demonstrated to have remarkable capabilities of hyaline cartilage formation and increased chondrocyte functions [42]. In a polyacrylamide-Fe_3_O_4_ magnetic nanocomposite hydrogel platform, an external magnetic field can temporally modulate the stiffness, which induces stem cell differentiation [43]. Viscoelasticity (stress relaxation) is another mechanical property influencing cellular behaviors [44]. PSCs and iPSC-derived organoids displayed increased lumen maturation and polarization when exposed to a faster stress relaxation rate by altering alginate's molecular weight (MW) or doping hydrazone [45,46]. Generally, these synthetic hydrogels are biologically inert compared to naturally derived hydrogels due to the insufficiency of endogenous biological components. Some biocompatible materials, such as biodegradable protein-loaded microspheres [47], can enhance the biocompatibility of synthetic hydrogels when added to matrices.

## 3 History of inner ear organoid research

**Fig. 2:**
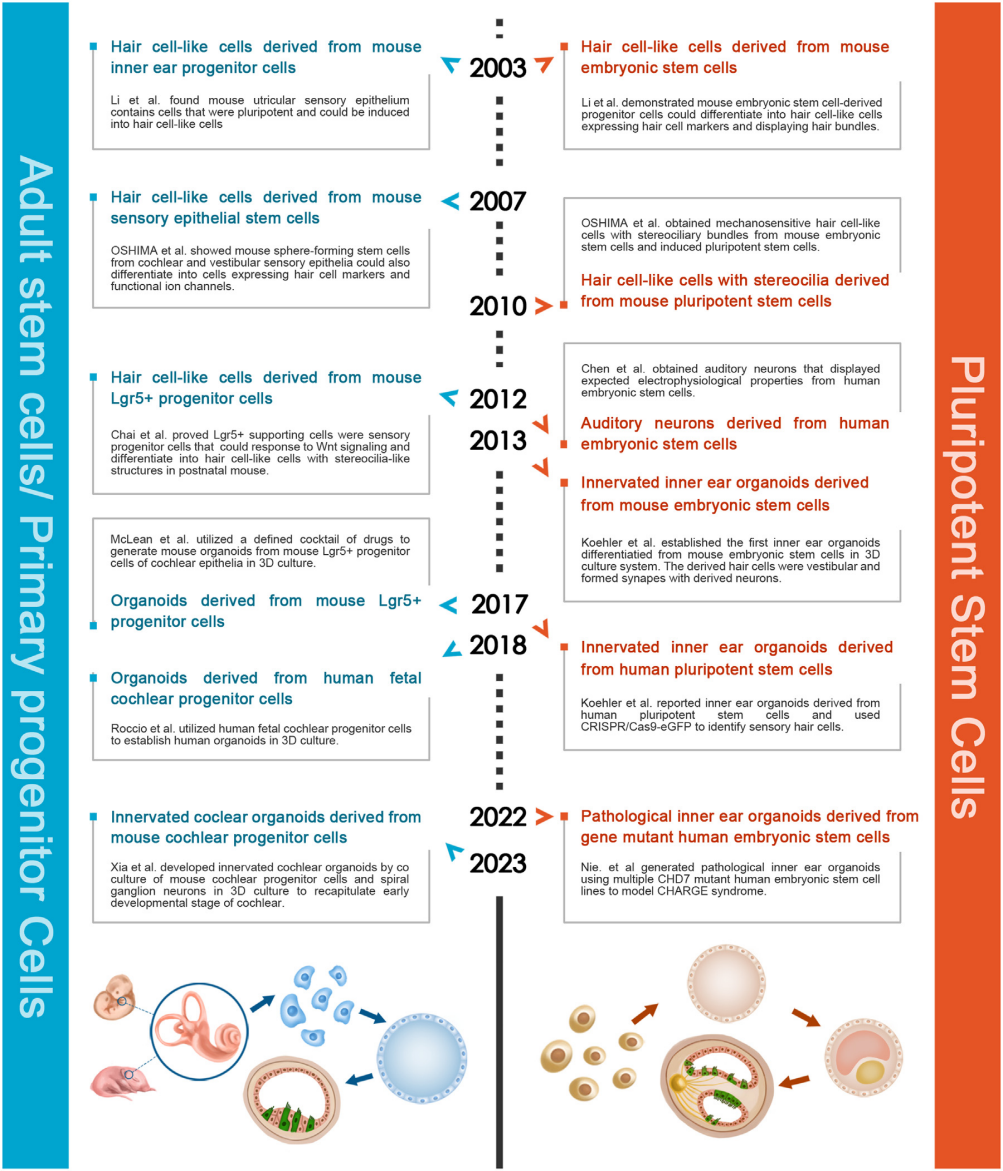
**Evolutionary history of inner ear organoid.** Milestones in the evolution of inner ear organoids derived from ASCs/PPCs and PSCs are listed on the left and right according to the timeline. Brief illustrations of protocols for generating these two types of organoids are provided, shown below in the same order.

Inspired by HCs and neurons derived from PSCs and discoveries in inner ear regeneration, researchers utilized 3D culture technology to differentiate murine and human PSCs into inner ear organoids consisting of HCs, neurons, and mesenchymal cells (Table 1). Additionally, murine neonatal and human fetal cochlear progenitors could be expanded as HC-containing organoids *in vitro*. The development of a coculture technique has made it possible to generate neuron-innervated cochlear organoids, thus forming an organoid model recapitulating the peripheral auditory circuit (Fig. 2).

**Table 1:** **Summary of PPC- and PSC-derived inner ear organoids**.

Original cell types	PPC-derived inner ear organoids	PSC-derived inner ear organoids
	PPCs	ESCs/iPSCs
Culture protocols	• Neonatal mouse cochlear progenitor cells were expanded with growth factors (EGF, bFGF, IGF) and a small molecule cocktail (CHIR, VPA, pVc, and 616452) for 7-10 days. Then, CHIR and LY were applied for another 14 days to induce hair cell differentiation [55,56]. • Neonatal mouse cochlear progenitor cells were expanded in culture medium with EGF, bFGF, IGF, CHIR, and LPA for 8 days to form organoid, then co-cultured with SGN explants by a staged combination of bFGF, BDNF, Shh, A83-01, RA, and LY for 30 days [16]. • Human fetal progenitor cells were expanded in medium containing EGF, bFGF, IGF, heparan sulfate, and CHIR for 2 weeks. Organoid differentiation was induced with LY and co-culture of EPCAM- cells for another 2 weeks [60].	• Murine ESCs were seeded in 2% Matrigel. BMP4 and SB were added on day 3 to induce non-neural ectoderm. bFGF and LDN treatment on day 4.5 induced preplacodal ectoderm. On day 8 of differentiation, aggregates were transferred to a floating culture to allow self-guided differentiation for the following 2 weeks [63]. • For human PSC induction, BMP4/ SB and bFGF/ LDN treatment were similar to that of murine ESCs. CHIR was added on day 8 to induce the formation of otic vesicles. On day 12, the aggregates were resuspended into Matrigel droplets. The droplet aggregates were moved to a floating culture on day 18 and CHIR was removed at this point. The aggregates could be maintained on an orbital shaker for up to 140 days [15].
Common features	• Matrigel provided 3D support in the culture system for both PPC- and PSC-derived inner ear organoids. • The organoids possess well-structured epithelial morphology, containing hair cell-like cells, stereocilia-like bundles, and even synapse-like structures. • Hair cells are in primary-level developing stages and displayed immaturity regardless of their origin.	
Unique features	• Recapitulating the cochlear epithelial structure, which can be innervated by neurons in an organoid-SGN explant co-culture system. • The HC-like cells resemble the pattern of native cochlear HCs. • Gene editing strategies could be applied to these organoids to model hereditary deafness.	• Comprising various cell types, such as mesenchymal, epithelial, and endothelial cells, while the proportion of HCs is relatively low. • ESCs/iPSCs-derived HCs display the properties of the primary vestibular HCs. • Somatic cells from patients can generate iPSC-derived organoids to model hereditary deafness.

CHIR, CHIR99021; VPA, valproic acid; pVc, 2-phospho-L-ascorbic acid; LY, LY411575; SB, SB-431542; LDN, LDN-193189.

### 3.1 Inner ear organoids derived from organ-restricted ASCs/PPCs

#### 3.1.1 Otic spheres derived from mammalian stem or progenitor cells

The generation of the neurospheres from the proliferative stem or progenitor cells inspired the research on otic stem cells [48]. Li et al. claimed that the sensory epithelium of the adult mouse utricle contains stem cells that retain sphere-forming ability, which was the first study to uncover the existence of inner ear stem cells [49]. A serum-free culture medium system with EGF and insulin-like growth Factor 1 (IGF-1) promoting sphere formation was established. Spheres were attached to differentiate into HC-like cells without growth factors. Oshima et al. modified the protocol by adding bFGF. They compared the sphere-forming ability of putative stem or progenitor cells resident in the vestibular, cochlear sensory epithelia, and spiral ganglion regions from postnatal Day 1(P1) and P21 [50]. The results showed that the capacity for sphere formation in the vestibule was the highest, while that of the cochlea sharply decreased during the first three postnatal weeks. It was demonstrated that otic spheres isolated from vestibular and cochlear sensory epithelia turned into HC-like cells, expressing the HC markers BRN3C, MYO7A, and ESPIN and exhibiting functional features similar to nascent HCs [49,50]. Subsequently, *Lgr5*, expressed in a subset of neonatal and mature cochlear supporting cells, was identified as the marker of cochlear epithelial progenitor cells [51]. Under Wnt signaling stimulation, Lgr5+ progenitor cells proliferated and developed into HCs [52,53]. Although Lgr5 was not expressed in the utricle after birth, exogenous damage induced its expression in the striolar region, and those Lgr5+ cells regenerated HCs through mitotic and transdifferentiation manners [54].

#### 3.1.2 Inner ear organoids derived from the murine cochlea

Thanks to the development of 3D culture technology, researchers were deeply engrossed in establishing inner ear organoids to replace spheres. Based on 3D Matrigel scaffolds, McLean et al. defined a culture system for Lgr5+ cochlear progenitor cells to grow into colonies and differentiate into HC-containing organoids with a combination of growth factors and compounds [55]. Importantly, activating Wnt and Notch signaling is required to promote the proliferation of Lgr5+ cochlear progenitor cells, while inhibiting Notch and activating Wnt is required to promote HC differentiation. Thus, Lgr5+ progenitor cells were exposed to the Wnt signaling activator CHIR99021(CHIR) and the histone deacetylase inhibitor valproic acid (VPA) [31] at the proliferation stage, and EGF, IGF, bFGF, 2-phospho-L-ascorbic acid, and 616452 were also included in the expansion medium. Later, the Notch signaling inhibitor LY411575(LY) and CHIR were combined to induce HC differentiation. This differentiation protocol produced a highly reproducible HC yield (> 25% of total cells), which was 67-fold greater than earlier approaches that merely used growth factors to culture progenitor cells [50]. The outer hair cell marker PRESTIN and the inner hair cell marker, vesicular glutamate transporter 3 (VGLUT3) were identified in HCs from the cultured organoids, and the active mechanoelectrical transduction channels were also detected through FM-1-43 staining. In addition, the same protocol also generated cochlear organoids from the inner ear cells of adult mice (P30 and P60), rhesus macaques, and the human inner ear. However, the efficiency was much lower than that obtained from neonatal mice. Using the expansion combination, Kubota et al. [56] found that the population of cells on the greater epithelial ridge (GER), a transient neonatal cell group located medial to the inner HCs and their surrounding supporting cells, has the most substantial potential to form organoids.

Since previous organoids contained only epithelial cells, our team recently developed neuron-innervated cochlear organoids with functional HC-spiral ganglion neuron (SGN) circuits [16]. By activating Wnt and YAP signaling with CHIR and lysophosphatidic acid (LPA), Lgr5+ cochlear progenitor cell-derived organoids were expanded for nearly 3 months under series passage. The expanded cochlear organoids were cocultured with dissected SGN explants in 3D Matrigel droplets. Multiple factors and combinations were screened to characterize a coculture system that balanced the differentiation of HCs and the outgrowth of neurites. After 30 days of differentiation, functional synapses were established between HCs and SGNs. Furthermore, coculture with SGNs increased the production of HCs within organoids significantly more than differentiation without SGNs.

#### 3.1.3 Inner ear organoids derived from the human fetal prosensory domain

Although rodents and humans share high homology (> 99%) in the genome [57], murine models exhibit significant differences from the human auditory system in inner ear development and regeneration [58,59]. Considering the current lack of human cell lines with otic identity, this dilemma has inspired researchers to build human inner ear organoids. Recently, Roccio et al. utilized EPCAM+ progenitor cells from the human fetal prosensory domain (PSD) region to generate inner ear organoids [60]. The 8-12-week postconception stage was chosen when the human cochlear PSD is at the postmitotic stage and HCs start to differentiate. Progenitor cells from the PSD region were sorted with the indication of CD271 and EPCAM and then expanded in a culture medium (containing EGF, bFGF, IGF, heparan sulfate, and CHIR99021) with 2% Matrigel. To promote progenitor cell-derived EPCAM+ organoid differentiation, LY411575 was added to replace the cocktail of the expansion stage and cocultured with EPCAM− cells in a semipermeable Transwell insert system. Organoids were formed with well-structured epithelial organization, which retained an average of 200 HC-like cells in each organoid. HC-like cells displayed typical HC morphology, expressed HC markers, and were able to take up aminoglycosides. No further advances were found in the human progenitor cell-derived organoids, which might be attributed to the difficulty of obtaining human-origin samples and strict ethical rules.

### 3.2 Inner ear organoids derived from ESCs/iPSCs

Apart from inner ear organoids derived from primary progenitor/stem cells, ESCs and iPSC cell lines serve as another source for generating organoids. When researchers induced ESCs/iPSCs into HCs 20 years ago, this breakthrough paved the way for continuously optimizing subsequent cultivation strategies to generate HCs and otic neurons. In 2013 and 2017, murine and human inner ear organoids were successfully established and applied to disease models in subsequent studies.

#### 3.2.1 HCs from ESCs/iPSCs in 2D culture

Li et al. first induced HC-like cells generated from mESCs through a stepwise protocol [61]. Combining EGF, IGF-1, and bFGF selectively enriched inner ear progenitor cells. Growth factor withdrawal then induced progenitor cells to differentiate into HC-like cells expressing multiple sensory epithelial markers. The ESC-derived progenitor cells were also injected into the otic vesicle of a chicken embryo to demonstrate their ability to integrate into developing inner ear sensory patches.

To optimize the previous induction protocol, HC-like cells with bundles from murine ESCs and iPSCs were reported in 2010. Those HC-like cells possessed electrophysiological characteristics similar to HCs *in vivo* [62]. In this study, IGF-1, the Wnt signaling inhibitor DKK1, and the TGF-β inhibitor SIS3 were applied to promote the formation of the anterior ectoderm while suppressing the induction of endo- and mesoderm. Subsequently, otic induction was guided by bFGF, resulting in the development of Pax2-positive otic progenitors. To further initiate HC generation, a layer of mitotically inactivated chicken utricle stromal cells was applied to the coculture. The HC-like cells expressed the typical HC markers MYO7a and ESPIN, bearing stereocilia and a kinocilium with similar architecture to native vestibular HCs.

#### 3.2.2 Inner ear organoids from mESCs/iPSCs in 3D culture

In addition to the guidance of soluble factors, the application of 3D culture with Matrigel was a breakthrough for inner ear organogenesis *in vitro* [63,64]. In 2013, Koehler et al. developed the first inner ear organoid from mESCs by stepwise induction in a Matrigel-based 3D culture system [63]. The combination of BMP and TGF-β inhibitor SB-431542 on Day 3 induced the formation of nonneural ectoderm without aberrant mesendoderm. Subsequently, reverse steps of inhibition of BMP signaling and activation of FGF signaling guided differentiation of the ectoderm toward presumptive otic placode expressing PAX2 and PAX8 at Day 8. Different from the endogenous Wnt signaling in the protocol [65], the Wnt agonist CHIR99021 was used in the subsequent study [66] to induce the formation of otic vesicles. At Day 20, epithelial cells of otic vesicles eventually developed into definitive HCs and supporting cells. Most of the derived HCs resembled vestibular Type II HCs based on cellular and cilia bundle morphology, tissue organization, specific marker expression, and electrophysiology properties. Unlike organoids derived from inner ear progenitor cells with a single epithelial unit, ESC/iPSC-derived organoids contain several cell types, ribbon synapses with extended neurites of stem cell-derived sensory neurons and synapse activities were also observed in newly generated HCs, suggesting the functional integrity of the 3D inner ear organoid [63,65].

#### 3.2.3 Inner ear organoids from hESCs/iPSCs in 3D culture

Human PSC-derived organoids are in high demand because they are more homologous to human tissue cells than murine organoids. Chen et al. exploited a protocol to induce otic cell generation from hESCs by FGF3 and FGF10 signals [20]. These otic placode-specifying factors could induce two distinct types of otic progenitors that differentiate into HC-like cells and auditory neurons. Transplantation of otic progenitors into a neuropathic deafness mouse model restored hearing function. In 2017, a stepwise induction established human inner ear organoids with sensory epithelia innervated by sensory neurons from hESCs. This process is highly similar to the generation of murine organoids, except that low FGF2 and CHIR99021 were added in the initial stage and induction of the otic formation stage, respectively [15]. Consistent with murine organoids, the electrophysiological properties of hPSC-derived HCs are similar to those of native sensory HCs and qualify as vestibular Type II HC phenotypes. Kurihara et al. designed a stepwise organoid protocol for differentiating hiPSCs into SGN-like cells [21]. After the generation of otic progenitors, trophic factors, including FGF2, EGF, IGF1, and WNT3A, were added to the 3D culture system to promote autonomous differentiation and maturation toward SGN-like cells. These SGN-like cells can be divided into type I and type II SGNs, which are similar to those of primary SGNs in protein expression profile, morphological characteristics, and electrophysiological properties. hiPSC-derived SGN-like cells in otic organoids recapitulate SGNs in situ and can be used as an experimental model of drug-induced neuropathy. Thus far, ESC/iPSC-derived HCs resemble the properties of primary vestibular HCs, while neurons induced from ESCs/iPSCs have properties similar to those of the cochlear.

## 4 Application of inner ear organoids

**Fig. 3:**
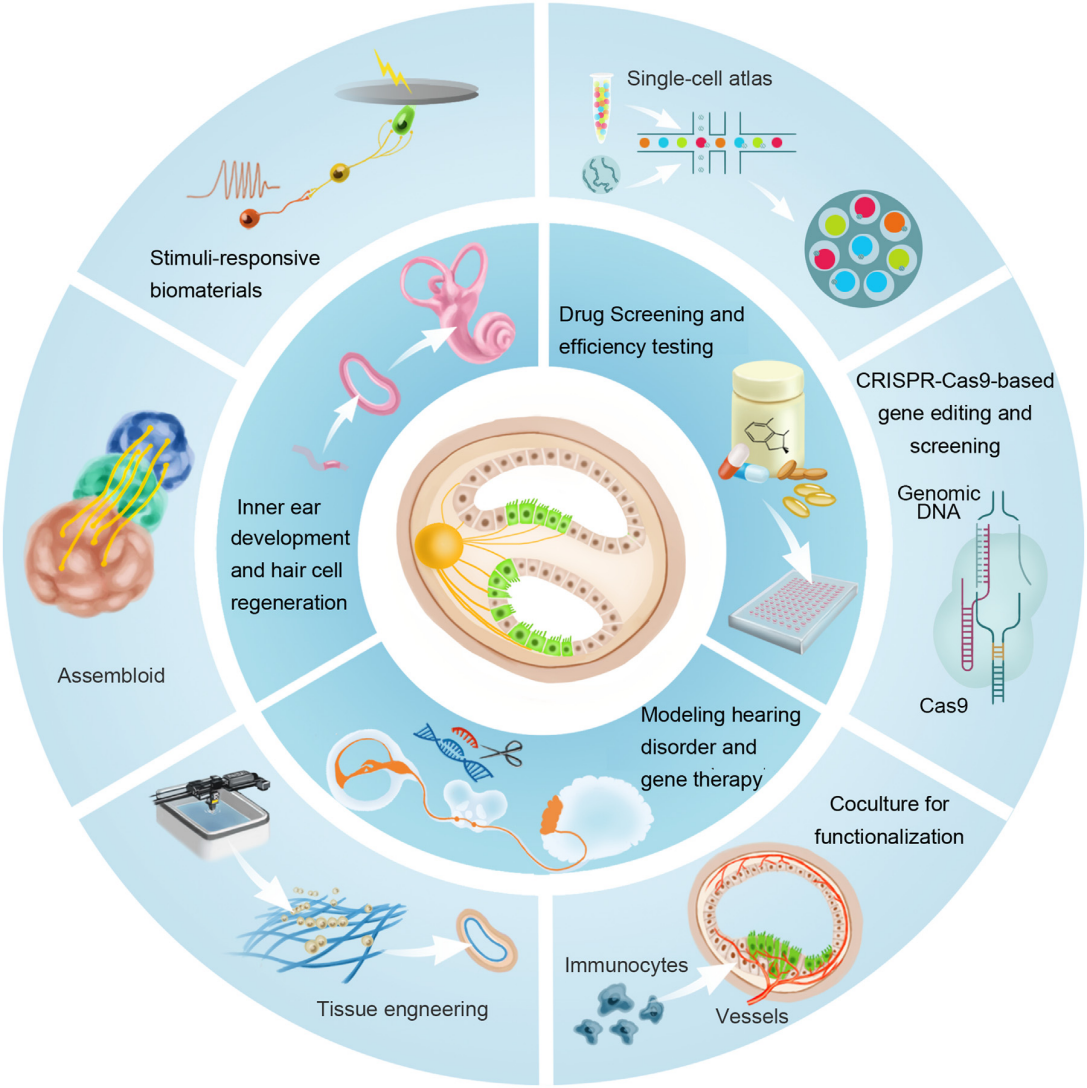
**The applications of inner ear organoids.** The inner ear organoids have been proposed for some meaningful applications (internal circle), For example, recapitulation of the inner ear development process for high-throughput drug screening and efficacy testing. Next-generation organoids of the inner ear can be ameliorated by emerging techniques and advanced materials (external circle). For example, tissue engineering provides three-dimensional tunable matrices and mechanic forces to induce expected organogenesis.

Hitherto, the development of inner ear organoids has led to its application in hearing research to study organ development, disease modeling, and drug screening (Fig. 3). Here, we describe the present and potential applications of inner ear organoids in various hearing sensory fields.

### 4.1 Inner ear development and hair cell regeneration

The inner ear development is a complex event of oriented differentiation, including successive formations of the preplacodal region in the ectoderm, otic placode, otic vesicles, and epithelia/spiral ganglia. Inner ear organoids are potent models for mimicking this developmental event, which is powerfully demonstrated by a recent report on the transcriptome features of developing inner ear organoids [16]. A series of evaluations were performed in this report on different stages of cocultured cochlear organoids, revealing that the HCs of the organoids could mimic the development pattern of the cochlear HCs up to P7 *in vivo*. The development process is guided through several delicate regulatory factors, such as TGF-β, FGF, BMP, Notch, and Wnt [67,68]. After taking advantage of inner ear organoids, more potential signaling pathways and genes involved in inner ear development were found, and these mechanisms provided possible clues for HC regeneration. DeJonge et al. found that Wnt signaling, indispensable in otic placode development *in vivo*, also drove the induction of the otic vesicles in ESC-derived organoids [66]. Precisely timed and dose-controlled Wnt agonist treatment promoted the induction of otic progenitor cells *in vitro* and further boosted the differentiation efficiency of HCs. Li et al. revealed that the RNA binding protein LIN28B reprograms supporting cell plasticity using cochlear organoids. LIN28B can promote cell type conversion from immature supporting cells/progenitors to HCs in a mTORC1-dependent manner [69]. In addition, the activin antagonist follistatin (FST) was recently confirmed to be another coregulator antagonizing TGF-β signaling to mediate supporting cell reprogramming and HC regeneration in organoid models [70]. Similar support of cell reprogramming was observed in the inner ear organoid when hypermethylated in cancer 1 (HIC1), a transcriptional repressor known to inhibit Atoh1 in the cerebellum [71], was knocked down and interacted with Wnt signaling to induce Atoh1 expression. Organoids offer a valuable platform for studying the development process and excavating strategies to manipulate the regeneration process.

### 4.2 Modeling hearing disorder and gene therapy

Generally, there are two approaches to modeling hereditary deafness *in vitro*: 1) transduction of deafness-associated mutations into wild-type cell lines by CRISPR-Cas9 and 2) induction from somatic cells of hereditary-deaf patients into iPSCs [72]. Tang et al. developed a mouse ESC-derived inner ear organoid model via CRISPR-Cas9, recapitulated type II transmembrane protease 3 (*TMPRSS3*) dysfunction, and detected early HC degeneration as a consequence of apoptosis [73]. Single-cell (Sc) RNA-seq analysis showed a disruption in intracellular homeostasis and extracellular matrix maintenance. Another pathogenesis model of the human inner ear organoid is CHARGE syndrome, which is caused by the embryonic lethal gene *CHD7* [74]. The CRISPR-Cas9-edited hESC cell line revealed that loss of *CHD7* led to dysregulation of early otic lineage genes and a partially drifted otic lineage identity, resulting in a complete absence of HCs and supporting cells. As a result of this study, various human *CHD7* mutant lines will be available for future drug testing and validation of gene therapy approaches specific to the human genome.

As well as recapitulating hereditary hearing loss, PSCs are an excellent tool for assessing the effectiveness of gene therapy and gene editing strategies. Based on the role of LIN28B in HC regeneration, as mentioned above, a double‐transgenic organoid model was established in which LIN28B expression was controlled by a tetracycline-on (TetOn) inductive system [69]. It was found that LIN28B overexpression could boost a progenitor-like state of supporting cells to regenerate HCs. Zhang et al. further validated the organoid model by assessing the induction efficiencies of different genetic engineering methods and demonstrated that TetOn induction had the highest efficiency with low cell apoptosis activity [75]. Although in the preliminary stage, these studies offered excellent references for clinical transplantation of gene therapy.

### 4.3 Drug screening and efficiency testing

In the past, researchers have used 2D cell culture and animal models such as mammals, birds, and amphibians to study sensorineural hearing loss and test potential protective drugs. However, compared to mammal models, 2D cell culture (such as the OC-1 proliferative cell line) and certain nonmammalian models (such as zebrafish) are more practical for drug screening and testing due to their effectiveness, accessibility, and affordability. Although the OC-1 cell line shows promising results in large-scale screening [76,77], it lacks the complex 3D structure and microenvironment of cochlear tissue. Additionally, it is genetically different from primary tissue and has distinct biological characteristics.

While primary cochlear tissues can be used for culture, it requires sacrificing many animals and the tissues can only survive for a limited time. Zebrafish models have been used for high-throughput genetic and drug screenings in the sensory system of the lateral line [78,79], but their distinct genome characteristics make them different from mammals in terms of susceptibility to drugs and reactivity to genes [80]. Additionally, zebrafish models have low screening effectiveness and strict feeding conditions. In contrast, mammal-derived inner ear organoids have more similar genomic characteristics to those *in vivo*, recapture 3D structures and have higher screening efficiency. Therefore, organoids are more advantageous than traditional OC-1 cell line culture and zebrafish models in drug screening. Recently, Liu et al. established a high-throughput platform with cochlear organoids derived from murine cochlear progenitors that achieved a 6,000-fold increase in sample output [81]. By screening over 1,000 FDA-approved small molecules, regorafenib, a VEGFR inhibitor, was identified as a potent small molecule for HC differentiation. Regorafenib was subsequently shown to mediate HC reprogramming and maturation in the cochlear pathological model.

Lenz et al. utilized Lgr5-positive cell-derived organoids to evaluate the effect of various epigenetic modifiers (e.g., DNA methylation) on inner ear development [82]. Cochlear organoids and animal models were employed to assess the impact of novel γ-secretase inhibitors (GSIs) and validate their efficacy on HC restoration *in vivo* [83].

## 5 Advanced prospects of construction and application of inner ear organoids

Although major breakthroughs have been made, there are substantial gaps in the application and clinical translation in organoids of the inner ear and organoids of large viscera. The application advancement was limited by diverse factors, such as the uncertainty of cell specialization and immaturity. Strategies to optimize organoid construction should be considered for continuous application and translation. In this section, we envisaged the combination of cutting-edge technologies to advance the application of these strategies. We proposed several promising approaches, such as tissue engineering, for optimizing next-generation inner ear organoids.

### 5.1 Limitations of current inner ear organoids

The major limitations of current inner ear organoids are that they are in primary-level developing stages and are immature. Due to the lack of effective differentiation cues and homogeneous nutrition supply, the current culture systems are insufficient for the inner ear organoid to resemble the adult inner ear system. Additionally, HC-like cells obtained in PSC-derived organoids are demonstrated to be single vestibular-like [15,65,84], and endeavors in directionally inducing cochlear HC organoids derived from PSCs are still needed. The specification of distinct cellular subtypes in inner ear organoids has also not been clarified, including HCs, and most nonsensory or mesenchymal cells that all play essential roles in regulating endolymphatic potential, providing mechanical forces to shape sense cells [85], [86], [87]. The immaturity of organoids hindered further functional studies and clinical applications. Thus, there is an urgent need for corresponding solutions using emerging techniques and materials, as mentioned below.

### 5.2 Single-cell atlas

Single-cell RNA sequencing (scRNA-seq) technology accurately identifies diverse cell types and provides insight into the essential genes related to development and disease. To explore the possible transcriptional heterogeneity and developmental cues, scRNA-seq analysis has been used as a valuable tool to access detailed information at single-cell resolution on cellular identities and the lineage trajectories of organoid cell types [16,88]. Organoid models resembling the pathological state of the inner ear can reveal the crucial role of mutant genes in inner ear development and provide fundamental information to validate genome-specific gene therapy [74].

Different animal species have varying regenerative abilities in their inner ear. While some lower vertebrates, such as chickens, have strong regenerative capabilities in their adult cochlea [89,90], hair cell loss after acoustic trauma in the mammalian inner ear is generally irreversible with limited spontaneous regeneration in vestibular sensory epithelia. Cochlear hair cells do not regenerate once damaged [88,91,92]. Recent studies on avian cochlea have shown promising cues for hair cell regeneration through single-cell RNA sequencing [93], [94], [95], which can potentially be tested on animal-derived organoids.

The technical popularity and optimized protocols of scRNA-seq also offer more opportunities to understand the overall characteristics of obtained inner ear organoids. In the future, combined with other single-cell multi-omics [96] and epigenetics [97], novel single-cell sequencing methods, including cellular indexing of transcriptomes and epitopes by sequencing (CITE-seq) and assay for transposase-accessible chromatin by sequencing (ATAC-seq) can reveal more valuable cues.

### 5.3 CRISPR-Cas9-based gene editing and screening

To obtain the pathological profiles of patients, gene mutation or deletion achieved by CRISPR-Cas9 editing technology could allow controllable and precise gene editing to recapitulate pathogenesis *in vitro*. Currently, CRISPR-Cas9 editing is widely utilized to model genetic-related carcinogenesis in organoids, such as colorectal cancer [98], gastric cancer [99], and ovarian cancer [100]. These organoids exhibited molecular, cellular, and even phenotypic characteristics of the corresponding mutated types. Moreover, by combining the advantages of the massive culture of organoids and unbiased gene detection of CRISPR-Cas9 editing, high-throughput screening of patient-specific vulnerable genes can be achieved, and potential therapeutic targets can be identified [101,102]. Meanwhile, optimized protocols and technologies have emerged. For example, CRISPR-Cas9-mediated homology-independent organoid transgenesis (CRISPR–HOT) enables more precise insertion of exogenous sequences and a higher rate of knock-in operation in different human organoids [103]. These studies all suggest that combining inner ear organoids and CRISPR-Cas9-based gene editing is promising for screening sensorineural hearing loss treatment strategies.

### 5.4 Tissue engineering

Similar to *in vivo* patterns, the self-organization of *in vitro* organoids can be influenced by local mechanical forces and various biochemical cues. Mechanical forces initiated by the physical properties of the extracellular matrix (e.g., shear stress from blood flow) and stress dynamics of adjacent mesenchyme (e.g., cell migration and proliferation) might drive organoid formation and specialization [104,105]. To mimic the mechanical forces, the facilitation of hydrogels with tunable mechanical properties that can control the microenvironment could be considered.

Comprehensive understandings of stress distributions in tissues allow for the designs of novel templated scaffolds or micropattern topography in line with physiological stress [106]. For example, stem/progenitor cells cultured in microbead chambers within a hydrogel could suffuse the internal surfaces of the chambers with an improved sphere formation capability [107]. At the differentiation stage, a mixture of type-I collagen and Matrigel hybrid matrix integrated to generate 3D laser-shaped microchannels allows intestinal organoids to self-organize into predefined shapes, specifically the tube-shaped epithelial structure with an accessible lumen and crypt-villus [108]. Bioprinting provides a technique to encapsulate organs and organoids within biomaterials by a layer-by-layer approach to create 3D biological geometries similar to that of native tissues with more precision [109,110]; for instance, 3D printed endothelialized microfiber scaffolds induced the generation of aligned myocardium of cardiovascular organoids [111]. Another advantage of tissue engineering is enabling uniform nutrition diffusion and separating the matrix microenvironment. Diffusion systems such as OCTOPUS [112] and microfluidic chips [113] can establish gradient homogenesis of diffusible regulatory factors and nutrition within organoids to allow expanded lifespan and specialization of cell types. Therefore, the utilization of biological scaffolds and 3D printing and other bioengineering might make inner ear organoids more structured, biomimetic, and mature.

### 5.5 Stimuli-responsive biomaterials

Besides endogenous biochemical cues and mechanical stress, organ morphogenesis can also respond to exogenous stimulation (light, electron, ultrasound, and magnetic fields) with engineered stimuli-responsive biomaterials [114]. The prominent advantage of these materials is that their stimulus can be precisely controlled both temporally and spatially, which might exert essential signals for organoid development. For instance, photoresponsive systems modulate covalent coupling/decoupling of bioactive cues and the physical topography of scaffolds to trigger the heterogeneous fate of stem cell populations [115]. Electrical, ultrasound, and magnetic field stimulations feature diverse release profiles of growth factors or drugs from the adhesive scaffold, enabling cell growth and nutrition supply. By comparison, light or ultrasound-responsive biomaterials are more capable of precisely regulating the local dynamics of drugs or cells. At the same time, electrical or magnetic response materials can maintain relatively uniform stimulations with high tissue penetration. Single stimuli or the integration of multiple stimuli can provide feasible differentiated approaches for organoid culture systems. Electrical stimuli-responsive materials can also be designed as stretchable mesh electronics that match the mechanical dynamics during organoid development to monitor electrophysiological properties in long-term culture [116].

### 5.6 Coculture for functionalization

A primary reason for premature organoids is the lack of diffusion pathways to supply essential nutrients and regulatory factors efficiently. Vascularization of organoids could improve maturation by maintaining fluid homeostasis and metabolic supply [117]. Similar to kidney organoids cocultured with endothelial cells, vascularization promoted the maturation and morphogenesis of perfused glomeruli to the capillary loop stage and mesenchymal proliferation [118]. Notably, when cocultured with cortical organoids, vascular cells were found to be enriched in the neurogenesis zone and promoted organoid maturation with various neural types (excitatory neurons, interneurons, astrocytes, and microglia) and spontaneous postsynaptic activities [119]. Meanwhile, the vascularized brain organoids also contained a blood-brain barrier (BBB)-like structure [120]. It is also promising for vascularized organoids to model the blood-labyrinth barrier (BLB) for drug targeting work, as BLB restricts the entry of most drug molecules [121]. The immune microenvironment of organs and the role of immune cells in the survival of inner ear cells has also gradually been revealed. For instance, macrophages can remove apoptotic cells or toxic metabolites by phagocytosis when stimulated [122]. Resident macrophages in the inner ear secrete various biofactors (e.g., growth factors), and the loss of SGNs can be induced by deficiency of the fractalkine (a macrophage chemoattractant) receptor after the death of HCs *in vivo* [123]. Immunomodulation has been demonstrated to be beneficial and applied in other organoids. For example, the coculture of PSC-derived microglia with brain organoids upregulated microglial surface proteins related to brain development to promote axon extension and dendrite arborization [124]. Because of the critical role of immunomodulation in the inner ear, it is necessary to load immune components into the inner ear organoid system.

### 5.7 Assembloid

The combination of various organoids recapitulating local areas into an assembloid allows insights into the interactions and crosstalk between these regions in an integral view. The emerging concept of assembloids was first proposed by Pașca et al. [125,126] for human cortical circuits. They integrated developed cortex organoids resembling the dorsal pallium and the subpallium and found that microcircuits were autonomously established between organoids. Their later study further demonstrated that cortex/spinal cord organoids could display concordant motor functions when assembled with skeletal muscle organoids [127]. Similar assembloids, including those of the digestive system [128], endometrium [129], and even entire embryo [130], were successively generated and applied in disease models. The integrality of the auditory pathway is extremely important for acoustic perception. Establishing a sensory auditory system composed of integrated peripheral and central auditory systems can broaden our insights into organogenesis, pathology, and functional coordination of intact auditory pathways. Although the auditory assembloid has a long way to go, it might be applied with microfluidic technology and bioscaffold guidance [131]. It deserves more research focus and possesses future application potential beyond an organoid.

## 6 Conclusion

Herein, we describe the developmental history and progress of the tissue-specific progenitor cell/PSC-derived inner ear organoids. To date, inner ear organoids have been partially applied to inner ear development/regeneration research, disease modeling, and drug screening. However, limitations, including low differentiation efficiency, immaturity, and unspecialized cell type, have hindered the extensive application of inner ear organoids. Endeavors should be made for designing next-generation inner ear organoids through various means. For example, emerging bioengineering technology, such as 3D-printing technology and microfluidic techniques, can be utilized to facilitate the optimization of the delicate structures of inner ear organoids and cell maturation. Coculture strategies to enable organoid vascularization and innervation to be functional could also be considered, as well as improving the performance of hydrogel scaffolds to create a better microenvironment. Hopefully, inner ear organoids will have brilliant prospects in the foreseeable future as a bridge between stem cell research and broader applications.

## Declaration of competing interest

The authors declare that they have no conflicts of interest in this work.
